# Observational evidence of legacy effects of the 2018 drought on a mixed deciduous forest in Germany

**DOI:** 10.1038/s41598-023-38087-9

**Published:** 2023-07-05

**Authors:** Felix Pohl, Ulrike Werban, Rohini Kumar, Anke Hildebrandt, Corinna Rebmann

**Affiliations:** 1 Helmholtz-Centre for Environmental Research - UFZ, Permoserstraße 15, 04318 Leipzig, Germany; 2 Friedrich Schiller University Jena, Institute of Geoscience, Burgweg 11, 07749 Jena, Germany; 3 German Centre for Integrative Biodiversity Research (iDiv) Halle-Jena-Leipzig, Puschstrasse 4, 04103 Leipzig, Germany

**Keywords:** Environmental sciences, Environmental impact

## Abstract

Forests play a major role in the global carbon cycle, and droughts have been shown to explain much of the interannual variability in the terrestrial carbon sink capacity. The quantification of drought legacy effects on ecosystem carbon fluxes is a challenging task, and research on the ecosystem scale remains sparse. In this study we investigate the delayed response of an extreme drought event on the carbon cycle in the mixed deciduous forest site ’Hohes Holz’ (DE-HoH) located in Central Germany, using the measurements taken between 2015 and 2020. Our analysis demonstrates that the extreme drought and heat event in 2018 had strong legacy effects on the carbon cycle in 2019, but not in 2020. On an annual basis, net ecosystem productivity was $\sim 16\,\%$ higher in 2018 ($\sim 424\,{\hbox {g}_{\text {C}}}\hbox {m}^{-2}$) and $\sim 25\,\%$ lower in 2019 ($\sim 274\,{\hbox {g}_{\text {C}}}\hbox {m}^{-2}$) compared to pre-drought years ($\sim 367\,{\hbox {g}_{\text {C}}}\hbox {m}^{-2}$). Using spline regression, we show that while current hydrometeorological conditions can explain forest productivity in 2020, they do not fully explain the decrease in productivity in 2019. Including long-term drought information in the statistical model reduces overestimation error of productivity in 2019 by nearly $50\,\%$. We also found that short-term drought events have positive impacts on the carbon cycle at the beginning of the vegetation season, but negative impacts in later summer, while long-term drought events have generally negative impacts throughout the growing season. Overall, our findings highlight the importance of considering the diverse and complex impacts of extreme events on ecosystem fluxes, including the timing, temporal scale, and magnitude of the events, and the need to use consistent definitions of drought to clearly convey immediate and delayed responses.

## Introduction

Forests are a major contributor to the terrestrial carbon sink^1,2^, which offsets approximately $25\,\hbox {to}\,30\,\%$ of human-caused carbon emissions^3^. However, extreme events such as floods, droughts and storms pose a major threat to the viability of natural systems and are responsible for much of the interannual fluctuations in the global carbon cycle^4,5^. Extreme drought and heat events in particular have severe negative impact on vegetation carbon uptake and storage capacity^6,7^, especially since they often occur simultaneously as so-called compound events^8,9^. Under the prevailing conditions of global warming, these events are expected to increase, both in duration and intensity in the future^10,11^. Understanding how those events alter forest carbon cycling is therefore critical for predicting future trends of the terrestrial carbon sink.

Continuous monitoring of ecosystems is an important source of information for studying the terrestrial carbon and water cycles. An effective method for this is the eddy covariance method, which can be utilized to derive the exchange of trace gases such as carbon dioxide and water vapor between the land surface and the atmosphere^12^. Eddy covariance measurements provide valuable insight into how the forest responds to changes in water availability, which in turn affects the forest’s ability to sequester carbon. Especially when measurements are conducted over several years, they allow to detect changes in the terrestrial carbon sink over time and attribute these changes to different drivers^13–15^. It also serves as a source of ground truth data for developing land surface models^16–19^ and has contributed to our understanding on the role of extreme events in the terrestrial carbon cycle^20–22^.

Droughts can reduce the CO$_2$ uptake of forests due to water scarcity causing plants to alter their stomata behavior to compensate for water loss^23^. Other non-stomatal plant processes such as changes in mesophyll conductance, Rubisco carboxylation capacity, and maximum electron transport rate^14,24,25^ can limit photosynthetic activity during droughts, but their interplay and implications for forest carbon cycling are unclear and subject of ongoing research^26,27^. The study of drought impacts is further complicated by the fact that not all responses are immediately apparent^28^. Delayed effects can arise from, for example, hydraulic damage and dieback^29–32^, shifts in carbon allocation^33–35^ and carbon depletion^36–38^, changes in nutrient cycling^39,40^ and reduced resistance to pest^41,42^.

The challenge of identifying lagged effects is separating the impact of previous causes from current conditions. Lagged effects have been mainly studied on an annual to monthly time scales using tree ring data^43,44^, remote sensing data^45^, carbon cycle models models^46^, or a mixture of those^47^, but studies on the ecosystem scale using daily eddy covariance measurements remain rare^28^. Legacy effects were also studied using regression analysis by identifying differences between predicted and actual ecosystem productivity in years following a drought event^47,48^. While regression models can be a powerful tool for understanding the functional relationships between ecosystem fluxes and potential drivers, attributing model errors to legacy effects remains tentative, as it may be that errors originate from other sources such as unobserved disturbances or model misspecification^49–51^.

Here we take a similar approach, but aim to incorporate information about past climate states directly into the analysis. We combine local measurements from the Hohes Holz (DE-HoH) research site, which provide detailed insights into the carbon and water cycles and associated meteorological measurements from 2015 to 2020, with a high-resolution dataset with daily standardized drought information aimed to support drought research at ICOS ecosystem sites by providing information on deviations from long-term climatology. Overall our goal here is to understand and analyse the (potentially delayed) responses of the 2018 drought and heat event on the terrestrial carbon cycle in a mixed deciduous forest in Germany. In particular, we aim to answer the following questions:

- (a) which impact did the extreme drought and heat event in 2018 have on carbon, water and energy exchange at the forest ecosystem Hohes Holz?
- (b) what are the temporal patterns at which aggregated climate conditions impact the ecosystem fluxes and
- (c) can incorporating drought information in statistical models improve predictions, especially during legacy years?

## Results and discussion

### Meteorology of the years of observation and relation to long-term climatology

**Figure 1:**
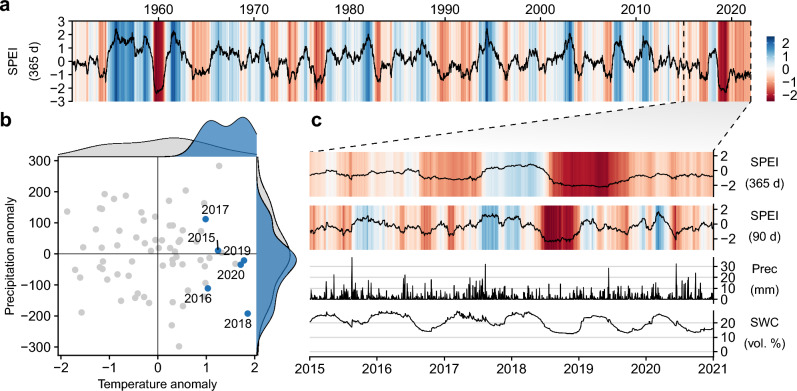
Climatic conditions of the observation years 2015–2020 in the context of long-term climatology (1950 - 2020) at the study site, (**a**) time series of 12-month SPEI from 1950 to 2020; the dashed vertical line indicates the start of the eddy covariance measurements, (**b**) annual values of precipitation and temperature of the six observation years at DE-HoH from 2015 to 2020 relate to the long-term data and (**c**) 3- and 12-month SPEI as well as local precipitation and soil water content at $50\,\hbox {cm}$ soil depth of the observation period.

During the observation period from 2015 to 2020, we generally observed that years were warmer than average, while annual precipitation varied. Putting these measurements in historical context (1950 – 2021)^52^, Fig. 1 shows that recent years have been mostly drier than usual, with the exception of the second half of 2017. Panel (a) shows a time series of the Standardized Precipitation-Evapotranspiration Index (365 days SPEI)^53^, with blue colours indicating periods that are wetter than usual and red colours indicating periods that are drier than usual. While wet and dry periods generally alternate, during the period of our measurements at DE-HoH the colour red dominates. The extreme drought event in 2018 stands out in particular, where standard deviations of below -2 indicate an occurrence probability of less than $2.3\,\%$. An event with similar severity was last recorded around 1960.

Examining panel (b) of annual temperature and precipitation anomalies reveals the underlying patterns of recent dry years. The empirical distribution of precipitation during the observation period (blue) roughly matches the distribution of historical precipitation data (grey). In contrast, annual temperatures since 2015 have all been in the range of 1 to $2\,^{\circ }\hbox {C}$ warmer than the long-term mean, suggesting that increased evapotranspiration due to warm conditions is having an additional negative impact on the water balance. Furthermore, 2018 stands out as the year with the highest temperature anomaly ($+1.86\,^{\circ }\hbox {C}$) and the third lowest precipitation sum ($301\,\hbox {mm}$), placing it at the extreme end of the joint distribution. This observation at the study site is consistent with other regions in Central Europe^11^. In comparison to the long-term annual values since 1950, the year of the last extreme drought event, 1959, had the lowest precipitation with $296\,\hbox {mm}$, but temperatures were only slightly higher than average by only $+0.4\,^{\circ }\hbox {C}$.

A detailed look at conditions during the measurement period is provided in panel (c) of Fig. 1, showing the temporal evolution of local precipitation patterns, soil water content (SWC) at $50\,\hbox {cm}$ soil depth and SPEI estimates corresponding to two aggregation time periods of 90 days and 365 days to reflect short-term (seasonal, affecting upper soil layer) and long-term (annual, affecting deeper soil layers) variability of governing weather conditions. Soil moisture usually follows a periodic pattern with a decrease at the beginning of the growing season and replenishing after the end of the growing season. Additionally, during the growing season, short-term rewetting due to large rain events occurs, particularly in the summer of 2015 and 2017, where persistent rain resulted in a strikingly small decrease in soil moisture. The SPEI time series both reflect the respective cold-wet and warm-dry conditions, wherein the persistence of these conditions depends on the aggregation period. The extreme event in 2018 as well as the exceptionally quick transition from wet to extremely dry conditions^54^ is well reflected in both SPEI timeseries. The short-term SPEI (90 d) indicates that drought conditions persisted until the end of the year, making 2018 the only year when soil moisture replenishment only started the following year. The beginning of 2019 consisted of a short wet period until another dry and warm, but not extreme growing season began. At the same time, the long-term SPEI (365 d) indicates persistent drought conditions, reaching its most extreme levels during spring and early summer 2019 and lasting until the end of summer 2019. The time series of SPEI for the two different aggregation periods highlight the difference between 2018 and 2019: while the meteorological conditions of 2019 were warm and dry, they do not themselves meet drought categories (i.e. SPEI (90 d) $> -1$). Due to the only moderate precipitation in winter the short-term drought was alleviated and soil water replenished in early 2019, while long-term drought (SPEI (365 d) $< -1$) continued throughout the summer of 2019.

### Fingerprint of drought on ecosystem fluxes

Eddy covariance data from 2015 to 2020 show that the forest acts as a carbon sink with an average net ecosystem productivity (NEP) of $\sim 362\,\hbox {g}\,\hbox {m}^{-2}\,\hbox {yr}^{-1}$. Annual and growing season values of eddy covariance and associated meteorological measurements are displayed in Table 1. In 2018, NEP was $\sim 16\,\%$ higher than in previous years before the drought. In contrast, NEP was $\sim 25\,\%$ lower in 2019 and returned to an average level in 2020. The only year that had a higher NEP than 2018 was 2017, which received most rainfall of the six years of observations with more than 600 mm annual precipitation. Table 1 indicates that these wet conditions in 2019 resulted in the lowest average vapor pressure deficit (VPD), air temperature (TA), and sensible heat flux (H). In contrast, the dry conditions in 2018 led to the highest VPD and H fluxes.

**Table 1:** Annual sums of net ecosystem exchange (NEE), gross primary productivity (GPP), ecosystem respiration (Reco) and precipitation (Prec) as well as annual averages of incoming short-wave radiation (SW$_\text {IN}$), latent heat flux (LE), sensible heat flux (H), net radiation (Rn), air temperature (TA), vapour pressure deficit (VPD) and length of the growing season derived from Phenocam pictures (cf. Fig. S1). The uncertainty estimates for the eddy covariance carbon fluxes are based on the bootstrapped u$^*$ threshold, while for the other variables it is the standard error of the annual mean.

		$\hbox {g}_{\text {C}}\,\hbox {m}^{-2}$			$\hbox {W}\,\hbox {m}^{-2}$		
	Year	NEP	GPP	Reco	H	LE	Rn
Full year	2015	309 (±32)	1675 ($^{+130}_{-158}$)	1366 ($^{+141}_{-210}$)	14.6 (±2.0)	43.3 (±1.9)	78.4 (±4.0)
	2016	354 ($^{+42}_{-3}$)	1724 ($^{+87}_{-165}$)	1370 ($^{+84}_{-207}$)	14.9 (±1.9)	44.0 (±1.9)	78.0 (±4.1)
	2017	439 ($^{+15}_{-49}$)	1629 ($^{+231}_{-48}$)	1190 ($^{+280}_{-63}$)	9.6 (±1.9)	46.3 (±2.1)	60.9 (±3.5)
	2018	424 ($^{+32}_{-50}$)	1572 ($^{+234}_{-72}$)	1147 ($^{+284}_{-109}$)	21.1 (±1.9)	42.2 (±1.8)	67.9 (±3.7)
	2019	274 ($^{+70}_{-84}$)	1291 ($^{+237}_{-106}$)	1016 ($^{+321}_{-176}$)	17.0 (±2.0)	41.9 (±1.8)	70.4 (±3.7)
	2020	372 ($^{+44}_{-32}$)	1694 ($^{+163}_{-159}$)	1322 ($^{+195}_{-202}$)	16.1 (±2.1)	44.8 (±1.8)	63.8 (±3.5)
Growing season	2015	544 ($^{+40}_{-0}$)	1607 ($^{+124}_{-145}$)	1064 ($^{+124}_{-184}$)	23.7 (±2.7)	65.4 (±2.4)	120.6 (±4.8)
	2016	578 ($^{+36}_{-10}$)	1614 ($^{+59}_{-156}$)	1036 ($^{+49}_{-192}$)	28.1 (±2.3)	66.5 (±2.4)	121.9 (±4.9)
	2017	622 ($^{+10}_{-41}$)	1552 ($^{+217}_{-42}$)	930 ($^{+258}_{-52}$)	18.9 (±2.6)	69.2 (±2.5)	95.7 (±4.2)
	2018	585 ($^{+26}_{-29}$)	1528 ($^{+227}_{-68}$)	944 ($^{+256}_{-95}$)	33.4 (±2.3)	59.1 (±2.2)	99.8 (±4.5)
	2019	498 ($^{+52}_{-70}$)	1253 ($^{+222}_{-92}$)	756 ($^{+437}_{-144}$)	30.2 (±2.4)	57.8 (±2.3)	101.5 (±4.2)
	2020	614 ($^{+39}_{-24}$)	1628 ($^{+154}_{-148}$)	1014 ($^{+177}_{-196}$)	29.7 (±2.8)	65.8 (±2.0)	101.2 (±4.0)

		$\mu \,\hbox {mol}\,\hbox {m}^{-2}\,\hbox {s}^{-1}$	$\,^{\circ }\,\hbox {C}$	$\hbox {mm}$	%	Days	
	Year	PPFD	TA	Prec	VPD	Length	
Full year	2015	237.5 (±9.4)	10.3 (±0.3)	550	4.0 (±0.2)		
	2016	222.1 (±8.9)	9.9 (±0.4)	389	4.2 (±0.2)		
	2017	201.1 (±8.3)	9.8 (±0.4)	610	3.8 (±0.2)		
	2018	272.3 (±10.7)	10.9 (±0.4)	302	5.9 (±0.3)		
	2019	250.5 (±10.0)	11.1 (±0.4)	471	5.3 (±0.2)		
	2020	255.7 (±10.0)	11.3 (±0.3)	478	5.2(±0.2)		
Growing season	2015	332.8 (±11.5)	14.4 (±0.4)	353	5.6 (±0.3)	205	
	2016	313.8 (±11.1)	14.7 (±0.4)	261	6.1 (±0.3)	207	
	2017	280.2 (±10.2)	14.2 (±0.3)	438	5.1 (±0.2)	215	
	2018	368.8 (±12.5)	16.1 (±0.4)	130	8.5 (±0.4)	227	
	2019	339.1 (±11.9)	14.9 (±0.4)	322	7.0 (±0.3)	230	
	2020	360.3 (±11.6)	15.5 (±0.3)	315	7.2 (±0.3)	211	

The annual values of the growing season carbon fluxes, shown in Table 1, highlight the severity of the drought in 2018. Only $43\,\%$ ($130\,\hbox {mm}$) of the total precipitation ($302\,\hbox {mm}$) fell during the growing season that year, compared to $67\,\%$ ($390\,\hbox {mm}$) in 2016, the second driest year of the study period. Nevertheless, net carbon uptake in 2018 was average over the observation period. In contrast, despite average precipitation and incoming radiation, 2019 had the lowest net carbon uptake. This suggests that the extreme heat and drought event in 2018 had a lasting impact on ecosystem fluxes in 2019. Both of these years also had significantly longer growing seasons, with an additional 19 days according to Phenocams estimates (see Supplement Fig. S1). Bastos et. al^55^ have shown that in Europe in 2018, as during the 2003 drought, in terms of annual carbon balance, high spring productivity offsets reduced carbon sequestration during the summer due to drought, similar to our research site. This highlight the need to consider potential impacts on legacy years, as consequences on carbon balance might not be prominent in the year of the heat wave itself.

**Figure 2:**
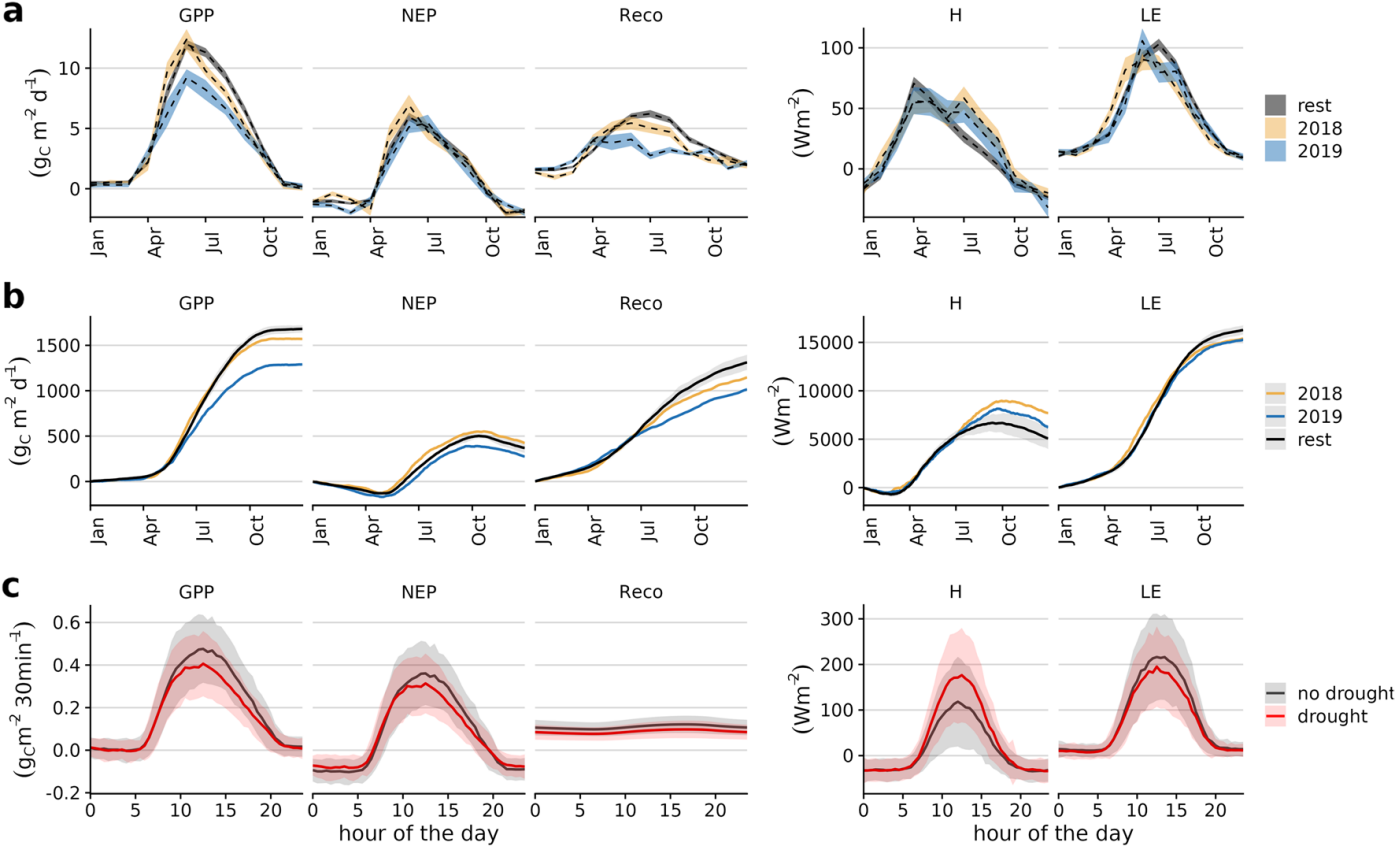
Time series of ecosystem fluxes net ecosystem productivity (NEP), gross primary productivity (GPP), ecosystem respiration (Reco), latent (H) and sensible heat (LE) at (**a**) monthly, (**b**) daily and (**c**) diurnal scale.

Fig. 2 shows how compounding dry and hot conditions affected ecosystem fluxes at seasonal to hourly time scales. On a monthly scale (panel (a)), net carbon sequestration in May and June was equal or even higher in 2018 than in other years, indicating that warm temperatures, clear sky and high radiation boosted gross primary productivity (GPP) until the water deficits most likely caused stomatal closure to regulate transpiration from July on^56^. While monthly averages of NEP were only slightly below average during the growing season in 2019, GPP and ecosystem respiration (Reco) were strongly reduced. The consequences on the carbon balance become especially apparent for 2019 when fluxes are summed up over the whole year as done in panel (b), where deviations from the usual annual progression of accumulated fluxes start notably in July.

Regarding the energy fluxes, sensible heat (H) usually peaks in April and decreases once the growing season begins, as vegetation activity alters the Bowen ratio^57^. Due to an early onset of the growing season, H is lower in April 2018 and 2019 compared to the average of the remaining years. Remarkable is also the increase of H in July 2018 which demonstrates how the drought decreased evapotranspiration, i.e LE relative to H. This behaviour of the Bowen ratio is known to appear under dry soil conditions^58–60^. LE shows a remarkable pattern in 2018, with above-average values through May, followed by the lowest monthly averages of the observation period during the summer months, giving an overall picture of a forward-shifted growing period. Sensible heat is also increased in summer 2019 to comparable levels to 2018 from July on, indicating re-occuring drought stress on the ecosystem^61^.

Panel (c) of Fig. 2 reveals the average diurnal timing of anomalies in the fluxes over the vegetation period. Strongest anomalies for the carbon fluxes occur during the afternoon, while H has strongest differences between drought and non-drought conditions during midday. Note that for visual reasons, we averaged the diurnal cycle for both short and long-term drought. This shows that despite temporarily increased photosynthesis in the early summer of 2018, over the entire drought period the drought had a negative impact on diurnal carbon sequestration.

**Figure 3:**
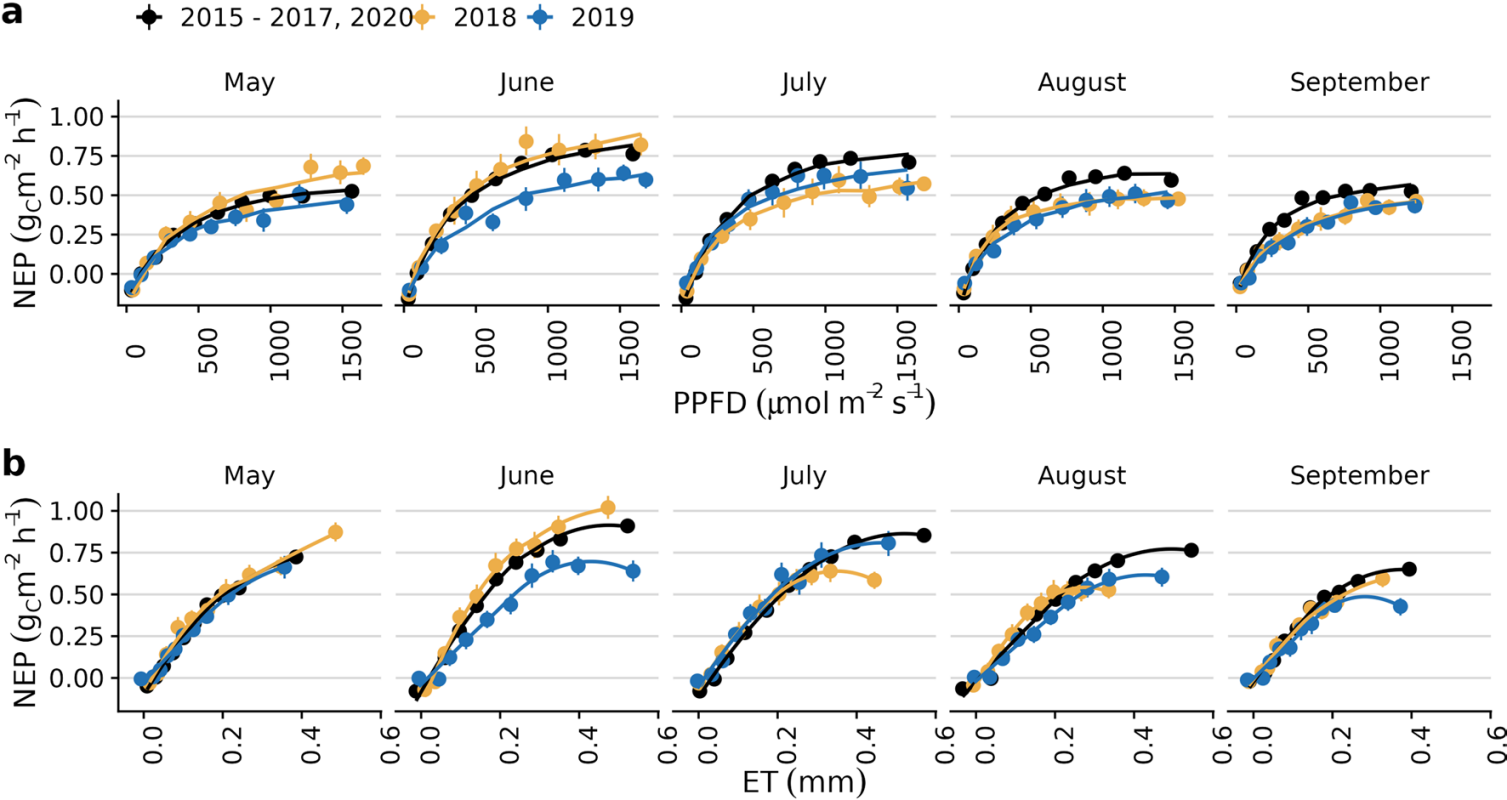
Relationship between net ecosystem productivity (NEP) and photosynthetic photon flux density (PPFD, **a**) and evapotranspiration (ET, **b**), respectively, plotted on a monthly basis and separately for 2018, 2019 and the other years. Curve in panel a is the Michaelis-Menten equation fitted as described in Falge^62^, while in panel b it is a local polynomial smoothing curve^63^.

**Table 2:** Parameters of Michaelis-Menten equation of Fig. 3a, fitted as described in Falge (2001)^62^. $\alpha$ is the ecosystem apparent quantum yield in $\upmu \,\hbox {mol}({\hbox {CO}_2}) / \upmu \,\hbox {mol}({\hbox {Photon}})$ and reference GPP is the ecosystem productivity in $\upmu \,\hbox {mol}_{\hbox {CO}_{2}}\,\hbox {m}^{-2}\,\hbox {s}^{-1}$ at light saturation of 2000 $\upmu \,\hbox {mol}\,\hbox {m}^{-2}\,\hbox {s}^{-1}$.

Month	$\alpha$			GPP_ref		
	2018	2019	others	2018	2019	others
May	0.059	0.050	0.061	21.19	15.45	18.80
June	0.088	0.056	0.089	26.78	19.19	26.32
July	0.078	0.067	0.091	19.07	18.51	25.76
August	0.103	0.055	0.094	17.07	16.19	22.49
September	0.054	0.044	0.094	15.17	15.08	18.80

The relationship between daytime CO$_2$ fluxes and PPFD or evapotranspiration, respectively, reveals the efficiency of the forest carbon sequestration under given light and water conditions. Net carbon sequestration per photon (Fig. 3a) respectively per water evaporated (Fig. 3b) was at average or even higher in May and June 2018, indicating the positive effect of the previous wet winter causing well-saturated soils in combination with the warm and sunny early summer of 2018. Yet, in July 2018, carbon sequestration efficiency drops. The parameters for the light response curve in Table 2 show that reference GPP (at 2000 $\upmu \,\hbox {mol}\,\hbox {m}^{-2}\,\hbox {s}^{-1}$) reached only 75 % of productivity in July and August 2018 compared with non-drought years. Even though soil water content replenished to some extent in 2019, light efficiency was still at similar low levels as in 2018. The relationship between carbon sequestration and evapotranspiration is very similar, except that it rises to near-normal conditions in July 2019 before dropping significantly again in August.

If taking the lower light use efficiency in panel (a) and water use efficiency in panel (b) as indications of stress, the low efficiencies in June and August 2019 could be interpreted as water stress. Water stress starting in July 2018 is in accordance with the soil moisture data. But although observed soil moisture down to $50\,\hbox {cm}$ and short-term SPEI (90 d) was near normal at the beginning of the growing season 2019, both light and water use efficiency were surprisingly low. This indicates a (potentially) lagged effect from the previous year, not related to the soil water content in the upper soil layers or short-term drought.

### Relationship between drought at various time scales and eddy covariance fluxes

**Figure 4:**
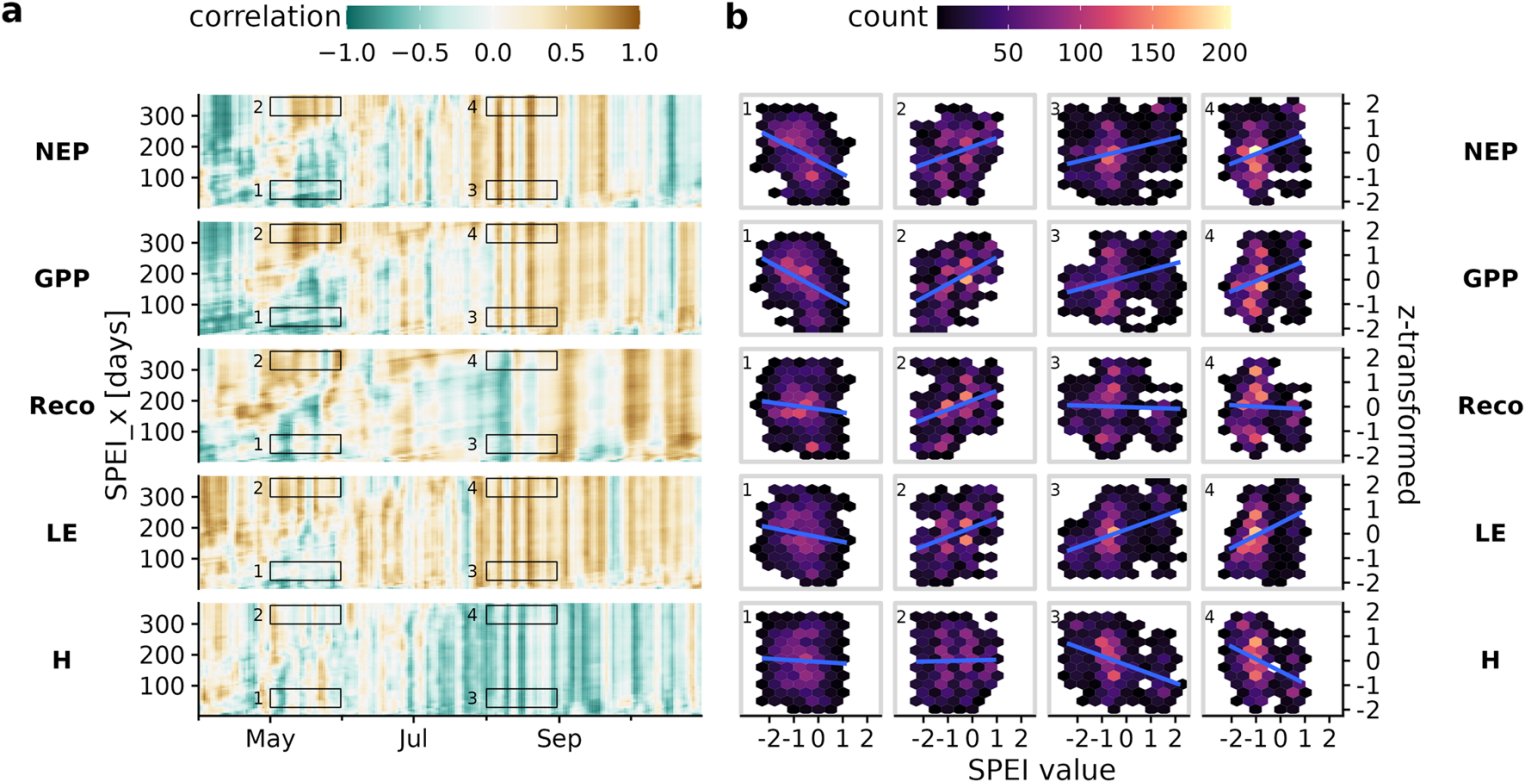
Time series of running correlation between between SPEI at aggregation scales from 5 to 365 days and standardized ecosystem fluxes (**a**). Panel (**b**) contains scatter plots of the z-transformed eddy covariance fluxes against the SPEI values of four selected areas from (**a**). (1) and (2) reflect the responses to short-term and long-term drought, respectively, at the beginning of the growing season, while (3) and (4) reflect the responses to short-term and long-term drought, respectively, in August. The blue line is a simple regression to illustrate the overall relationship between the individual windows.

To better understand how time scales of droughts and their timing during the growing season affect the ecosystem fluxes, we looked at their relationship with SPEI for multiple aggregation periods. Panel (a) in Fig. 4 shows the multi-year relationship between SPEI at aggregation times from 5 to 365 days and daily standardized ecosystem fluxes during the growing season. The relationship was examined for each day of the year using a rolling correlation with a window of five days (to avoid low statistical power^64,65^) and standardized observations over the six observational years. For each ecosystem flux, four areas (indicated with boxes in Fig. 4a) are highlighted where the standardized measurements are plotted against the SPEI values during that period (Fig. 4b).

The pattern of temporal correlation is similar among both carbon fluxes and energy fluxes, with the sign reversed between latent and sensible energy. Especially for the second half of the growing season, ET is mostly positively correlated across all aggregation scales of SPEI, while H is mostly negatively correlated. This implies that the Bowen ratio is more sensitive to climatic conditions during the second half of the growing season which can most likely be explained by the availability of soil moisture^59^. Soil moisture availability is contingent on weather patterns during the summer. Fig. 4 shows that the Bowen ratio in the latter part of the summer was influenced by both short-term and long-term weather conditions.

The patterns of carbon fluxes show primarily a negative correlation at the beginning of the growing season in May for shorter aggregation periods of SPEI, which transitions to a positive correlation for longer periods. Because of the large daily fluctuations in ecosystem fluxes, the strength of the relationship is also variable from day to day, as the drought indices exhibit much higher temporal persistence. It therefore makes sense to look at the correlations together over larger time periods as well, which we do exemplary for May and August in panel (b) of Fig. 4; and thereby reflecting the conditions during the start and mid of the growing season, respectively. While the wide dispersion of the data reflects the multiple influencing factors, the trend line (blue) shows whether or not there is a relationship with the drought indices when viewed over the entire period of the respective area. Here the slope of the regression line corresponds roughly to the average correlation of the four selected areas.

Area 1 corresponds to SPEI with aggregation periods of 30 to 90 days in May, reflecting short- to midterm deviations from normal climate conditions. Especially NEP and GPP are notably negatively correlated, meaning that warm and dry spells before and at the beginning of the growing season enhance carbon sequestration. Note that during the start of the growing period in the spring season, despite moderately drier conditions (compared to climatology), soils in forest hold sufficient moisture (in absolute term) to adequately support their phenological development. Thus, the warm conditions during spring and clear sky further enhance vegetation development. However as these conditions deplete the soil moisture storage during spring, without an adequate supply of water through rainfall in the subsequent summer, the forest productivity declines^66^. In contrast, the long-term drought index in May, shown in area 2, is negatively correlated with carbon fluxes as well as with ET. Areas 3 and 4 correspond to short and long-term drought in August. NEP, GPP, and LE are negatively affected regardless of aggregation time, which in turn favors an increase in H. Reco seems to be relatively unaffected by drought, as indicated by a slope close to zero. But a deeper look at the time series in panel (a) shows that the period included in the sample covers times with both positive and negative correlation, which averages out when aggregated over longer periods.

### Estimating forest productivity under extreme conditions

We found that carbon sequestration of the forest ecosystem was noticeably impacted during the extreme heat and drought of 2018, and that low light and water use efficiency persisted into the year 2019 (Fig. 3). Although this can be an indicator of prolonged drought stress^67,68^, reduced carbon sequestration in 2019 could also result from unfavorable weather conditions. To understand how much ecosystem productivity could be expected given the hydro-meteorological conditions, we use a regression model (i.e. Restricted Cubic Spline; RCS regression) to estimate GPP as a function of Photosynthetic Photon Flux Density (PPDF) and Soil Water Content (SWC) in $50\,\hbox {cm}$, and compare it against a model that includes additional information on drought conditions (SPEI_90 and SPEI_365). The presence of legacy effects may be indicated by a notable difference in the performance of the two regression models as only one of the models has information on past climate conditions. We chose GPP rather than NEP as response variable because GPP is more directly attributed to changes in predictors than NEP, which is the balance between GPP and Reco and would thus require additional consideration of changes in autotrophic and heterotrophic respiration.

**Figure 5:**
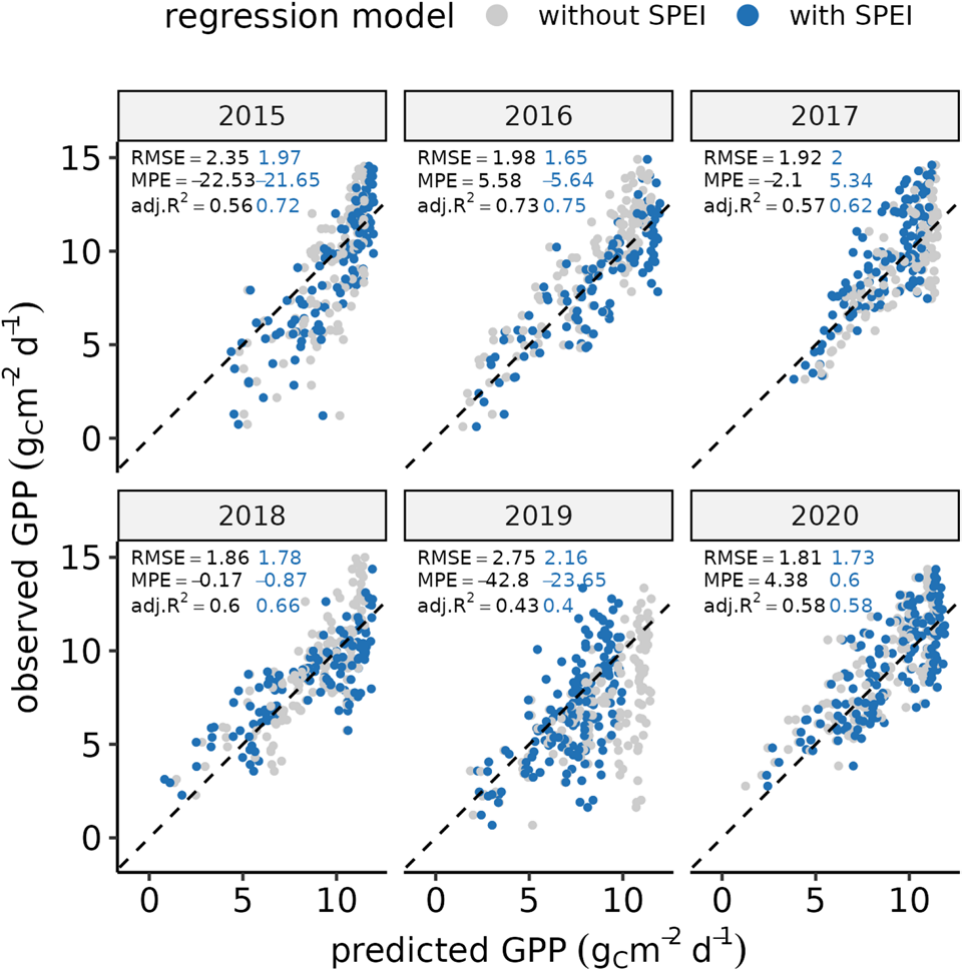
Scatter plot of observed vs. predicted GPP using restricted cubic spline regression for years 2015 to 2020. Predictions are based on soil water content (SWC) and photosynthetic photon flux density (PPFD) (gray dots) and considering additionally SPEI_90 and SPEI_365 (blue dots). Results are plotted for each year separately and with root mean squared error (RMSE), mean percentage error (MPE) and R2 adjusted for number of predictors.

Overall, the RCS model with SPEI had higher explanatory power (adj. R$^2$ = 0.64) compared to the model based solely on SWC and PPFD (adj. R$^2$ = 0.54). The Akaike information criterion (AIC) of the complex model (AIC = 3572) was substantially lower than that of the simple model (AIC = 3786), indicating that the additional complexity is worth considering in terms of the increase in explanatory power. A comparison of actual versus predicted GPP for each year shows that in 2019, the mean percentage error (MPE) was -42.8 % (Fig. 5). This represents a significant overestimation of actual productivity, which is not seen in any other year. Including SPEI in the model reduces the MPE to -23.65 %, but the adj. R$^2$ of 0.4 is still the lowest of all observation years. In 2020, there are no significant differences between the two models, indicating that the observed productivity can be explained by the model just as well as in other years without drought, and that GPP was not affected by legacy effects.

Other studies in Central Germany have found decreases in tree growth in 2019 in a floodplain forest^69^, but stronger legacy effects in 2020 than 2019 in an old-growth and more diverse forest (DE-Hai)^48^. Yu et al.^48^ used a random forest regression model to quantify legacy effects, but focused on residuals rather than the difference in explanatory power. It is unclear whether the difference in lagged responses between this study site (DE-HoH) and more diverse forest site (DE-Hai;^48^) is due to differences in forest structure or can be attributed to the different regression approaches. Both forests have European beech (Fagus sylvatica L.) as dominant species, but DE-HoH consists also of 46% (basal area) sessile oak (Quercus petraea (Matt.) Liebl.), while DE-Hai has 28% of ash (Fraxinus excelsior). Some studies attribute higher sensitivity of ash to environmental changes than oak^70,71^, but whether this explains the differences in lagged responses remains unclear and requires further research.

**Figure 6:**
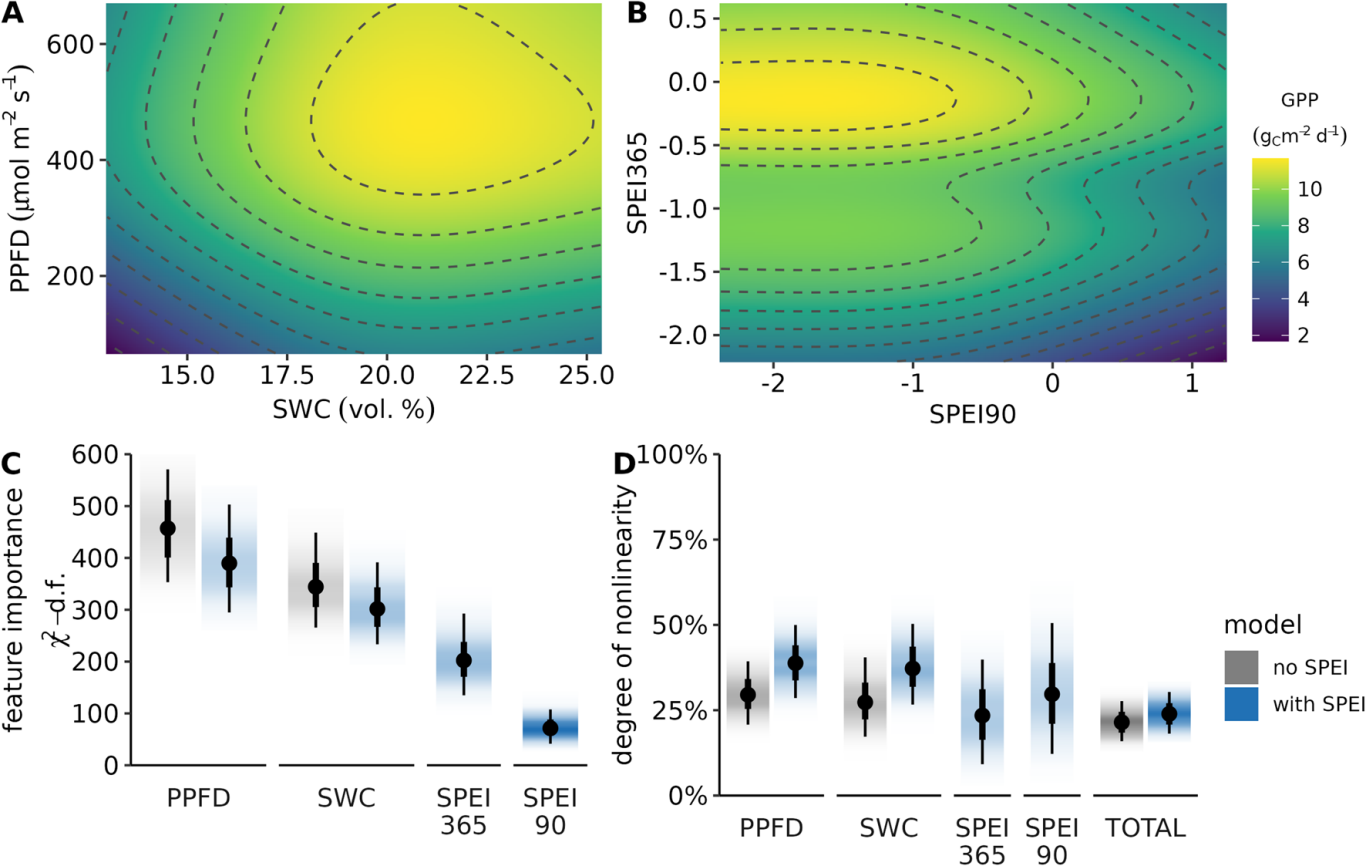
Global interpretation of the restricted cubic spline regression models with gross primary productivity (GPP) as response. Bivariate partial dependence plot between photosynthetic photon flux density (PPFD) and soil water content in 50 cm (SWC) (**a**) and SPEI_90 and SPEI_365 (**b**). Feature importance (**c**) is defined by $\chi ^2$ minus degrees of freedom and degree of nonlinearity (**d**) is defined as sum of squares explained by the splines.

Parametric models with splines can account for non-linear responses. While machine learning algorithms can outperform classical statistical methods, they often come at the cost of lack of interpretability. In this study, we minimized the complexity of the model to allow more robust evaluation of the model results. Optimal daily gross primary productivity of our forest ecosystem was found to occur at soil water content (SWC) of approximately 20–22.5 vol. % and daily average photosynthetically active photon flux density (PPFD) of $400\,-\,600\,\upmu \,\hbox {mol}\,\hbox {m}^{-2}\,\hbox {s}^{-1}$ (Fig. 6a). Based on the results of the RCS model, we notice that both long-term drought and short-term wet spells reduce forest productivity (Fig. 6b). This is in line with this region being both potentially energy and water limited. Energy limited ecosystems can benefit in the short run from increase in available energy^72^, as indicated by low SPEI (90 d). But longterm drought can shift them to water limitation and therefore SPEI (365 d) around zero are preferred.

The partial dependence plots in panel (a) and (b) of Fig. 6 need to be interpreted in conjunction with the relative importance of each feature (Fig. 6c). The majority of the additional contribution from the more complex model comes from the Standardized Precipitation-Evapotranspiration Index (SPEI) with a time window of 365 days, while SPEI with a time window of 90 days has only a small influence. PPFD is the most important variable, explaining most of the day-to-day variability, followed closely by SWC. Both were also identified as most dominant in other data-driven studies on forest productivity^73–75^.

The inclusion of SPEI as a covariate slightly reduces the relative importance of PPFD and SWC as dominant predictors. In environmental data, it is common for predictors to be correlated with each other to some extent. Therefore, the introduction of additional predictors is expected to affect the partial sum of squares. However, the small reduction in the chi-squared statistic for PPFD and SWC suggests that most of the information contributed by the additional predictors is independent and not multicollinear. The RCS model also allows for the attribution of explained variance to the linear and spline terms in the equation (Figure 6d). Overall, the splines accounted for approximately $25\,\%$ of the explained variance, while the linear relationship accounted for the remaining $75\,\%$.

### Possible reasons for legacy effects and limitations of the study

The Standardised Precipitation-Evapotranspiration Index provides useful insights in two important ways. Firstly, it serves as a versatile multi-index due to its flexible parameterisation in terms of accumulation time. This allows different time periods to be considered and both short and long term anomalies to be assessed. Secondly, our study provides some evidence that the long-term SPEI can be an important tool for improving estimates of carbon uptake under legacy situations where it operates as a surrogate for otherwise challenging-to-measure responses at the leaf-level. However, the study is not sufficient to make a general recommendation, as the short observation period and the limitation to one site mean that the transferability to other sites and extreme events has not yet been tested. The use of a standardised drought index dataset^76^ would make it possible to identify potential common patterns of extreme events in similar ecosystems in a large-scale, multi-site study.

Some possible mechanisms, which could explain the legacy effects, include hydraulic damage to the trees, which directly limits radial growth by decreasing photosynthetic capacity, and indirectly by preferential allocation of photoassimilates to replenish non-structural carbohydrate stores^28,77^. This could partly explain the low carbon fluxes at the beginning of the growing season in the year following the extreme event, which is in line with other studies reporting delayed spring phenology in the subsequent year^78^. Measurements of gas exchange at leaf level, water potential and hydraulic conductivity would be required to advance the understanding of the mechanisms leading to the lagged effects, also in relation to the different responses of the individual species^28^. Furthermore, separate measurements of autotrophic and heterotrophic respiration would allow to differentiate between the response patterns of the individual respiration fluxes^79^. However, these measurements are very laborious and would need to be maintained for many years to measure both pre- and post-drought situations, so they may be more feasible in experimental and controlled environments.

Other factors may involve the loss of deep water reservoirs, which are challenging to observe but known to be linked to forest die-off^80^. In fact, our water level measurements show that the level dropped significantly in spring 2019 following the 2018 drought, and the actual depth could not be determined since then due to inaccessibility (cf. Fig S2). It is reasonable to assume that this has implications on the water support at the research site as oak e.g. are known to be able to reach deep water resources^81^. However, the specific rooting depth at our research site remains unknown, so the effect of the drop in water level is unclear.

Research of the mechanisms behind legacy effects is still in its infancy, with most evidence coming from dendrochronological analysis^28^. This has led to a range of drought effects being found in temperate and boreal forests, but the results of these studies are diverse as the effects can be related to a variety of factors. Furthermore, the recent spread of eddy covariance data and drought studies has shown that during drought, there is a decoupling of tree-ring legacy effects from gross primary productivity^28,82,83^. For attributing lagged responses in forest fluxes with certainty, future studies will need comprehensive measurements from before and after drought situations. Additionally, studies need to strategically integrate experimental research with model development to facilitate robust scaling from individual to ecosystem and remote sensing levels.

## Conclusion

Extreme drought events pose a significant threat to forests and can disrupt ecosystem processes for multiple years. However, the rarity of such extreme events limits the opportunities for targeted ecosystem studies. Additionally, our process understanding of the impacts of drought on ecosystem functioning is still limited, making it difficult to accurately model the future trajectory of the terrestrial carbon cycle. In the face of global warming, it is crucial that we gain a deeper understanding of the consequences of extreme droughts in order to effectively model and mitigate their impacts. To accurately assess the effects of an extreme drought event on ecosystem fluxes, we used a combination of eddy covariance and complementary hydrometerological measurements from a deciduous forest from 2015 to 2020, along with the standardized precipitation-evapotranspiration index. In this context, it is important to consider the extreme event from two view points: The 3-month SPEI (90 days) illustrates the extreme water deficit in summer 2018, while summer 2019 falls within the expected long-term climate variability. In contrast, the 12-month SPEI (365 days), identifies 2019 as an extreme drought year based on the aggregated water deficit. This emphasizes the importance of using standardized indices for accurately communicating and interpreting legacy effects to avoid misunderstandings about actual and lagged responses to extreme events^76^.

In 2018, the combination of a well-saturated soil and an early start of the growing season enhanced carbon sequestration. Despite severe drought stress later in the summer, the carbon uptake for 2018 was above average. This is consistent with other studies on the effects of drought on European forests, that have shown that the carbon balance during extreme event years may not be as severe due to compensatory effects within the annual cycle. However, in 2019, carbon fluxes decreased to record lows, likely due to a combination of legacy effects from the previous year at the start of the growing season and soil moisture stress in the later summer. Regression analysis indicates that such reduced carbon uptake could not simply be explained by the current hydrometeorological conditions given by radiation and soil moisture measurements. The use of the standardized precipitation-evapotranspiration index (SPEI) as a covariate in regression modeling yet improved estimates of forest productivity under extreme conditions.

While further information such as dynamics of deep water reservoirs would be beneficial to identify the causes for lagged responses, we could demonstrate that using SPEI with different aggregation times as co-variate in regression modelling could improve estimates of forest productivity, making it a promising tool to improve our understanding of ecosystem carbon balance under extreme conditions. While carbon fluxes returned to normal levels in 2020, it is too early to conclude that there will be no lasting impacts from the extreme drought event. These impacts can persist for several years and require longer measurements and investigation to fully understand drought effects on ecosystem behaviour.

## Materials and methods

### Site description

The study area is part of the mixed deciduous forest ’Hohes Holz’ in the region of the Magdeburger Boerde in Central Germany ($52^{\circ }$ 05’ N, $11^{\circ }$ 13’ E, $200\,\hbox {m}$ above sea level). Climate in the study area is subatlantic-submontane with a mean annual temperature of $9.1^{\circ }$ C (climatic period 1981-2010, station Ummendorf of the German Weather Service), mean minimum daily temperature in the coldest month (January) of $0.7\,^{\circ }\,\hbox {C}$ and mean maximum of the warmest month (July) of $18.3\,^{\circ }\,\hbox {C}$. Annual mean precipitation was 563 mm during the climatic period 1981-2010, while annual precipitation during the investigated period measured locally at the site ranged from $301\,\hbox {mm}$ in 2018 and $610\,\hbox {mm}$ in 2017 (see Table 1). The forest stand is located in a mainly municipal forest area of about $15\,\hbox {km}^{2}$, managed by regional forestry. Within a fenced area of about 1 ha, intensive measurement equipment related to the carbon, water and energy cycles of the forest ecosystem was established since 2013, including an eddy covariance tower for measurements of trace gas fluxes between the forest ecosystem and the atmosphere.

The fenced area is composed of European beech (*Fagus sylvatica* L.), and sessile oak (*Quercus petraea* (Matt.) Liebl.) as dominant species (38 % and 45 % of total basal area, respectively) with accompanying hornbeam (*Carpinus betulus* L., 13 %) with 245 trees in the enclosure. Tree height and diameter at breast height were 24.0 ± 10.5 m, and 0.34 ± 0.21 m for *Fagus*, 29.6 ± 3.1 m and 0.47 ± 0.11 m for *Quercus*, and 17.7 ± 5.6 m, and 0.21 ± 0.07 m for *Carpinus* in 2020. Additional selected tree plots (CPs) were investigated according to the ICOS sampling design^84^, partly located outside the fenced area. Within the fenced area only trees in danger were harvested or fell during storms since 2011 and the surrounding area has thus a lower stocking degree due to regular thinning. There were no further treatments of plants or trees on the research site. Within these plots, beech and oak are also the dominant species (41 % and 46 % of total basal area, respectively), accompanied by hornbeam (10 %), sycamore (*Acer pseudoplatanus* L., 2 %) and birch (*Betula pendula* Roth, 1 %). Inventory data outside the fenced area are mainly from field inventories performed during winter 2017 / 2018. The bedrock is Pleistocene loess (Weichsel), with Haplic Luvisols and Stagnic Gleysol as predominant soil type. Soil texture at 0–20 cm depth is 3.0 ± 1.8 % sand, 87.1 ± 2.1 % silt, and 10.0 ± 2.2 % clay, with a pH-value of 8.0.

The study site ’Hohes Holz’ was established since 2013 within the framework of the TERENO-project as part of the Central German Lowland Observatory^85^ (https://www.tereno.net/) managed by the UFZ. Since the beginning of 2019, the station fulfills all required instrumentation and sampling procedures according to the ICOS ecosystem standards for class 1 stations^86,87^ (https://www.icos-cp.eu/observations/ecosystem). In addition to the ICOS requirements, several continuous and campaign-based measurements are performed, most of them related to the water balance of the forest ecosystem. Only those considered for the present analysis are detailed below. The station is well equipped with line power and internet access, such that all continuous data are transferred to institutional sftp on a daily basis.

#### Eddy covariance measurements

Continuous flux measurements with the eddy covariance (EC) technique are performed in 49 m height on the scaffolding tower since July 2014 using an ultrasonic anemometer (CSAT-3, Campbell Scientific Inc., Logan, UT, USA) and an open-path infrared gas analyser (LI-7500, LiCor Inc., Lincoln, NE, USA) for carbon dioxide (CO$_2$), water vapour (H$_2$O), sensible heat (H) and momentum ($\tau$) fluxes^88,89^. The sonic anemometer is directed to west-south-west according to the main wind direction prevailing in the area. High-frequency raw data are acquired with a CR3000 data logger (Campbell Scientific Inc., Logan, UT, USA) and collected, pre-processed and archived with the EDDYMEAS data acquisition module of Eddysoft^90^. Sampling frequency for wind components, sonic temperature and CO$_2$ and H$_2$O concentrations is 20 Hz. Since spring 2016 an additional EC system according to ICOS standards (GILL HS-50, Gill Instruments Ltd., Lymington, UK and LI-7200, LiCor Inc., Lincoln, NE, USA) was installed on the tower in the same height until Oct. 2018 and was moved to 45m height thereafter.

#### Storage fluxes

CO$_2$ and H$_2$O concentrations are measured along a vertical profile from 0.1 m above the soil surface up to 49 m height by sucking the air through equally long and heated tubings to a valve system that provides the air sequentially to a LI810A gas analyzer (LiCor Inc., Lincoln, NE, USA). The air of each of the 9 levels was analyzed for CO$_2$ and H$_2$O for 60 s before switching to the next level until a change of the system was done to fulfill ICOS-standards (June 14, 2018). Since this change, 3 additional levels were added (0.1 m, 0.4 m and 10 m) and the time sampled per level was reduced accordingly.

#### Meteorological and hydrological variables

The station is equipped with sensors on the tower for atmospheric variables as well as in the tower surrounding for soil variables. The main variables are short- and long-wave radiation components of both hemispheres, photosynthetic photon flux density, air temperature, air humidity, air pressure, precipitation, soil heat flux, soil temperature and soil moisture (see tab. 3). Those data are replicated in different heights along the tower or with depth in the soil, respectively and are sampled at a frequency of 0.05 Hz and aggregated in 10 min values by the data loggers (CR3000 or CR1000, Campbell Scientific Inc., Logan, UT, USA). Table 3 contains further details regarding measurement location, abbreviations, sensor types and manufacturers.

**Table 3:** Main sensors for hydro-meteorological variables measured at the ecosystem station ’Hohes Holz’.

Variable name	Variable description	Sensor type	Manufacturer	Depth/height (m)	Unit
NEE	net ecosystem exchange of CO$_2$	CSAT3, LI-7500RS	Campbell Scientific Ltd., LI-COR Biosciences	49	$\upmu \,\hbox {mol}\,\hbox {m}^{-2}\,\hbox {s}^{-1}$
H	sensible heat flux	CSAT3	Campbell Scientific Ltd.	49	W m$^{-2}$
LE	latent heat flux	CSAT3, LI-7500RS	Campbell Scientific Ltd., LI-COR Biosciences	49	W m$^{-2}$
u$^{*}$	friction velocity	CSAT3	Campbell Scientific Ltd.	49	m s$^{-1}$
CO$_2$	CO$_2$-concentrations along vertical profile for change of CO$_2$-storage	LI-840A	LI-COR Biosciences	49	$\mu$mol mol$^{-1}$
Prec	precipitation	tipping bucket 5.4032.35.008	Thies Clima	50	mm
SWC_a, SWC_b	soil moisture profiles a and b	CS616	Campbell Scientific Ltd.	0.1 - 0.8	%
airpres	air pressure	Setra 278	Setra Systems	49	hPa
Rn, SW_IN, SW_OUT, LW_IN, LW_OUT	net radiation, short and long wave radiation components	CNR4	Kipp & Zonen	49	W m$^{-2}$
PPFD	photosynthetic photon flux density	LI190R	LI-COR Biosciences	49	$\upmu \,\hbox {mol}\,\hbox {m}^{-2}\,\hbox {s}^{-1}$
G	soil heat flux	HFP01, HFP01SC	Hukseflux	0.05	W m$^{-2}$
TA	air temperature	HMP155	Vaisala	49	$^\circ$ C
RH	air relative humidity	HMP155	Vaisala	49	%
TS_a, TS_b	soil temperature profiles a and b	Model 107	Campbell Scientific Ltd.	0.1 - 0.8	$^\circ$ C

### Data processing

Flux computation from high frequency (20 Hz) raw data of the CSAT-3 / LI-7500-EC-system is performed with the EddyPro$\circledR$ software^91^ [LI-COR-Biosciences, 2017] with commonly used settings such as block averaging, Webb correction and planar fit for 4 sectors^92^. Subsequent post-processing steps such as estimating the u*-threshold, gap-filling and flux partitioning for net ecosystem exchange of CO$_2$ (NEE) are performed with the REddyProc open source software package^93^ after adding the CO$_2$-storage change from the profile data^94^. Thresholds for u* are estimated with the moving point method^95^ using data where SW_IN <  10 W m$^{-2}$. Bootstrapping is used to estimate the distribution of the u* threshold, and all subsequent processing is performed for the median quantile, while we report the uncertainty range in Tab. 1 using the $5\,\%$ and $95\,\%$ threshold. Gaps in NEE, air temperature and vapor pressure deficit (VPD) were filled using the marginal distribution sampling method^96^ with default variables and margins (SW_IN $50\,\hbox {W}\,\hbox {m}^{-2}$, TA $2.5\,^{\circ }\,\hbox {C}$ and VPD $5.0\,\hbox {hPa}$). NEE was partitioned into gross primary productivity (GPP) and ecosystem respiration (Reco) using both, the nighttime NT^96^ and daytime DT^97^ approach. Both approaches rely on the assumption that NEE measured at night is essentially ecosystem respiration (Reco), yet the NT-method is based on using fitting the Lloyd and Taylor equation^98^ and the DT-method is based on fitting a rectangular hyperbolic light-response curve^62^. We repeated all statistical analyses with partitioned fluxes of both methods (not shown) and found that the choice of partitioning method did not notably affect the analysis, so we only present results from the NT method.

### Phenocam

Pictures of the canopy were taken multiple times per day with a Stardot NetCam SC5 (Stardot Technologies, Buena Park, CA), which is located in $45\,\hbox {m}$ height in westerly direction on the same tower as the eddy covariance system. Using the ’phenopix’ R-package^99^ we performed the following steps to extract information about the phenological state of the ecosystem: For each picture and a fixed region of interest (ROI), the RGB channel values were extracted and the relative greenness (G$_{CC}$) of each image was calculated^100^. Derived G$_{CC}$ values were filtered for low illumination (G$_{CC}<0.2$), outliers^14^ and for change in scene illumination^101^. Afterwards, the filtered timeseries of G$_{CC}$ was fit to a double logistic sigmoid function^102^. Finally, the phenological transition dates were estimated using local extrema in the rate of change of curvature of the fit^102,103^. To estimate the uncertainty of the transition dates, the fit is repeated 100 times to randomly-noised data using the residuals between the original model fit and the observed data. The resulting fit is visualized in Supplementary Fig. S1. For 2015, the Phenocam images could not be analyzed using the described method due to incorrect color settings, so the derived phenological states were estimated from the images using expert knowledge.

### Standardized precipitation evapotranspiration index

To objectively quantify the drought at the research site, we used the Standardized Precipitation Evapotranspiration Index (SPEI)^104^. The SPEI takes into account both changes in temperature, i.e. potential evapotranspiration, and precipitation when evaluating water conditions. Studies have demonstrated that the response of vegetation to water conditions can vary significantly, with some effects being observed within a few months and others taking several years to manifest^105,106^. The SPEI can be used to study vegetation response across different timescales due to its capability of considering different aggregation periods. We used a dataset tailored to the ICOS sites with daily temporal resolution and constructed based on the E-OBS^52^ dataset. For transparency, we state that the authors of this study are also part of the authors of the dataset. For a detailed description of the methodology used to create the drought indices, we refer to the data descriptor^76^.

### Statistical analysis

We used regression analysis to investigate the relationship between GPP and potential predictors. Predictors were selected according to the equation of photosynthesis, which states that both water and photons are needed to absorb $\hbox {CO}_{2}$ from the atmosphere. Consequently, measurements of Photosynthetically Active Photon Flux Density (PPFD) were used as a predictor for light energy and upscaled soil water content measurements from $50\,\hbox {cm}$ (SWC_50) were used as a predictor for water availability. Additionally, we tested, whether drought indices could be useful predictors for including climatological effects into the models.

We use restricted cubic spline (RCS) regression to account for potential non-linear relationships. Cubic spline regression is basically a series of piecewise cubic polynomials in which the number of pieces is defined by so-called knots and constructed so that the function is smooth. They are restricted to be linear for $x<k_{min}$ and $x>k_{max}$ to optimize their behaviour in the tails^107^. The equation has the form of:

1 $$\begin{aligned} g(y) = \beta _0 + \beta _1x + \sum ^{k-1}_{i=2} \beta _i \cdot C_i(x), \end{aligned}$$

where *g* is a link function and $C_i(x)$ is the cubic component^108^. It has been shown that the position of the knots is not very sensitive^109^ so default quantiles can be used. In practice, a number of 3 – 5 knots is usually used^109,110^.

We used Akaike’s Information Criterion (AIC) to find optimal number of knots and to compare the overall performance of the regression models. AIC is a measure of relative quality of statistical models that takes into account both best fit and complexity of the model. Due to the latter it is also suited to compare models with different complexity, i.e. amount of parameters, to identify whether the incremental complexity is worth it. AIC is calculated as follows:

2 $$\begin{aligned} AIC = 2k - 2ln(\hat{L}) \end{aligned}$$

where k is the number of parameters in the model and L is the maximized value of the likelihood function of the model. A lower AIC is considered to be a better trade-off between model fit and degrees of freedom spent compared to a model with higher AIC.

We calculated $\chi ^2$ to determine feature importance of the predictors^110^:

3 $$\begin{aligned} \chi ^2 = \hat{\beta }_{S}^\top \widehat{\Sigma }_{S}^{-1}\hat{\beta }_S \end{aligned}$$

where S is a set of terms associated with the sub-model tested, $\beta _S$ is the corresponding subset of coefficient estimates and $\widehat{\Sigma }_{S}$ is their covariance matrix. The sub-model is a version of the full model without the predictor in question. This is equivalent to perform a F-Test on the significance of a predictor multiplied by the degrees of freedom, and the resulting $\chi ^2$ statistic is here the feature importance. Likewise, we expressed the degree of non-linearity of a predictor as the ratio between its splines’ partial sum of squares and the partial sum of squares of both, the linear and the splines term of that predictor^109^.

## Supplementary Information

Supplementary Information.

## Data Availability

The datasets generated during and analysed during the current study as well as code to reproduce all results and figures are available in the zenodo repository, https://doi.org/10.5281/zenodo.7638744.

## References

[1] Pan Y (2011). A large and persistent carbon sink in the World’s forests. Science.

[2] Keenan T, Williams C (2018). The terrestrial carbon sink. Annu. Rev. Environ. Resour..

[3] Le Quéré C (2009). Trends in the sources and sinks of carbon dioxide. Nat. Geosci..

[4] Zscheischler J (2014). A few extreme events dominate global interannual variability in gross primary production. Environ. Res. Lett..

[5] Friedlingstein P (2022). Global Carbon Budget 2022. Earth Syst. Sci. Data.

[6] Sippel S (2018). Drought, heat, and the carbon cycle: A review. Curr. Clim. Change Rep..

[7] Piao S (2019). The impacts of climate extremes on the terrestrial carbon cycle: A review. Sci. China Earth Sci..

[8] Zscheischler J (2020). A typology of compound weather and climate events. Nat. Rev. Earth Environ..

[9] Zscheischler, J., van den Hurk, B., Ward, P. J. & Westra, S. Chapter 4 - Multivariate extremes and compound events. In *Climate Extremes and Their Implications for Impact and Risk Assessment, 59–76* (eds Sillmann, J. *et al.*) (Elsevier, 2020). 10.1016/B978-0-12-814895-2.00004-5.

[10] Spinoni J, Vogt JV, Naumann G, Barbosa P, Dosio A (2018). Will drought events become more frequent and severe in Europe?. Int. J. Climatol..

[11] Hari V, Rakovec O, Markonis Y, Hanel M, Kumar R (2020). Increased future occurrences of the exceptional 2018–2019 Central European drought under global warming. Sci. Rep..

[12] Foken, T., Aubinet, M. & Leuning, R. The Eddy Covariance Method. In *Eddy Covariance* (eds Aubinet, M. *et al.*) 1–19 (Springer Netherlands, 2012).

[13] Kato T, Tang Y (2008). Spatial variability and major controlling factors of CO2 sink strength in Asian terrestrial ecosystems: Evidence from eddy covariance data. Glob. Change Biol..

[14] Migliavacca M (2011). Semiempirical modeling of abiotic and biotic factors controlling ecosystem respiration across eddy covariance sites. Glob. Change Biol..

[15] Rogger J, Hörtnagl L, Buchmann N, Eugster W (2022). Carbon dioxide fluxes of a mountain grassland: Drivers, anomalies and annual budgets. Agric. For. Meteorol..

[16] Williams M (2009). Improving land surface models with FLUXNET data. Biogeosciences.

[17] Verbeeck H (2011). Seasonal patterns of CO2 fluxes in Amazon forests: Fusion of eddy covariance data and the ORCHIDEE model. J. Geophys. Res..

[18] Fisher RA, Koven CD (2020). Perspectives on the future of land surface models and the challenges of representing complex terrestrial systems. J. Adv. Model. Earth Syst..

[19] Bahrami B (2022). Developing a parsimonious canopy model (PCM v1.0) to predict forest gross primary productivity and leaf area index of deciduous broad-leaved forest. Geosci. Model Dev..

[20] Yi C, Pendall E, Ciais P (2015). Focus on extreme events and the carbon cycle. Environ. Res. Lett..

[21] Rebane S, Jõgiste K, Põldveer E, Stanturf JA, Metslaid M (2019). Direct measurements of carbon exchange at forest disturbance sites: A review of results with the eddy covariance method. Scand. J. For. Res..

[22] Kannenberg SA, Bowling DR, Anderegg WRL (2020). Hot moments in ecosystem fluxes: High GPP anomalies exert outsized influence on the carbon cycle and are differentially driven by moisture availability across biomes. Environ. Res. Lett..

[23] Daszkowska-Golec A, Szarejko I (2013). Open or Close the Gate:Stomata action under the control of phytohormones in drought stress conditions. Front. Plant Sci..

[24] Egea G, Verhoef A, Vidale PL (2011). Towards an improved and more flexible representation of water stress in coupled photosynthesis-stomatal conductance models. Agric. For. Meteorol..

[25] Zhou S-X, Prentice IC, Medlyn BE (2019). bridging drought experiment and modeling: Representing the differential sensitivities of leaf gas exchange to drought. Front. Plant Sci..

[26] Rogers A (2017). A roadmap for improving the representation of photosynthesis in Earth system models. New Phytol..

[27] Gourlez de la Motte L (2020). Non-stomatal processes reduce gross primary productivity in temperate forest ecosystems during severe edaphic drought. Philos. Trans. R. Soc. B.

[28] Kannenberg SA, Schwalm CR, Anderegg WRL (2020). Ghosts of the past: How drought legacy effects shape forest functioning and carbon cycling. Ecol. Lett..

[29] McDowell NG (2013). Evaluating theories of drought-induced vegetation mortality using a multimodel-experiment framework. New Phytol..

[30] Anderegg WRL (2013). Drought’s legacy: Multiyear hydraulic deterioration underlies widespread aspen forest die-off and portends increased future risk. Glob. Change Biol..

[31] Hartmann H (2018). Research frontiers for improving our understanding of drought-induced tree and forest mortality. New Phytol..

[32] Venturas MD, Todd HN, Trugman AT, Anderegg WRL (2021). Understanding and predicting forest mortality in the western United States using long-term forest inventory data and modeled hydraulic damage. New Phytol..

[33] Trugman AT (2018). Tree carbon allocation explains forest drought-kill and recovery patterns. Ecol. Lett..

[34] Hasibeder R, Fuchslueger L, Richter A, Bahn M (2015). Summer drought alters carbon allocation to roots and root respiration in mountain grassland. New Phytol..

[35] Aaltonen H, Lindén A, Heinonsalo J, Biasi C, Pumpanen J (2017). Effects of prolonged drought stress on Scots pine seedling carbon allocation. Tree Physiol..

[36] Galiano L, Martínez-Vilalta J, Lloret F (2011). Carbon reserves and canopy defoliation determine the recovery of Scots pine 4 yr after a drought episode. New Phytol..

[37] Galiano L, Martínez-Vilalta J, Sabaté S, Lloret F (2012). Determinants of drought effects on crown condition and their relationship with depletion of carbon reserves in a Mediterranean holm oak forest. Tree Physiol..

[38] McDowell NG (2022). Mechanisms of woody-plant mortality under rising drought, CO2 and vapour pressure deficit. Nat. Rev. Earth Environ..

[39] Houle D, Lajoie G, Duchesne L (2016). Major losses of nutrients following a severe drought in a boreal forest. Nat. Plants.

[40] Schlesinger WH (2016). Forest biogeochemistry in response to drought. Glob. Change Biol..

[41] Jactel H (2012). Drought effects on damage by forest insects and pathogens: A meta-analysis. Glob. Change Biol..

[42] Simler-Williamson AB, Rizzo DM, Cobb RC (2019). Interacting effects of global change on forest pest and pathogen dynamics. Annu. Rev. Ecol. Evol. Syst..

[43] Anderegg WRL (2015). Pervasive drought legacies in forest ecosystems and their implications for carbon cycle models. Science.

[44] Szejner P, Belmecheri S, Ehleringer JR, Monson RK (2020). Recent increases in drought frequency cause observed multi-year drought legacies in the tree rings of semi-arid forests. Oecologia.

[45] Zhang T (2023). Response of ecosystem gross primary productivity to drought in northern China based on multi-source remote sensing data. J. Hydrol..

[46] He W (2018). Large-scale droughts responsible for dramatic reductions of terrestrial net carbon uptake over north america in 2011 and 2012. J. Geophys. Res..

[47] Wu X (2018). Differentiating drought legacy effects on vegetation growth over the temperate Northern Hemisphere. Glob. Change Biol..

[48] Yu X (2022). Contrasting drought legacy effects on gross primary productivity in a mixed versus pure beech forest. Biogeosciences.

[49] Rao P (1971). Some notes on misspecification in multiple regressions. Am. Stat..

[50] Hibbs DA (1973). Problems of statistical estimation and causal inference in time-series regression models. Sociol. Method..

[51] Hutcheon JA, Chiolero A, Hanley JA (2010). Random measurement error and regression dilution bias. BMJ.

[52] Cornes RC, van der Schrier G, van den Besselaar EJM, Jones PD (2018). An Ensemble Version of the E-OBS Temperature and Precipitation Data Sets. J. Geophys. Res..

[53] Pohl F (2022). Long-term daily hydrometeorological drought indices, soil moisture, and evapotranspiration for ICOS ecosystem sites. Sci. Data..

[54] Shah J (2022). Increasing footprint of climate warming on flash droughts occurrence in Europe. Environ. Res. Lett..

[55] Bastos A (2020). Impacts of extreme summers on European ecosystems: A comparative analysis of 2003, 2010 and 2018. Philos. Trans. R. Soc. B.

[56] Buckley TN (2019). How do stomata respond to water status?. New Phytol..

[57] Duveiller G, Hooker J, Cescatti A (2018). The mark of vegetation change on Earth’s surface energy balance. Nat. Commun..

[58] Herbst M (1995). Stomatal behaviour in a beech canopy: An analysis of Bowen ratio measurements compared with porometer data. Plant Cell Environ..

[59] Gu L (2006). Direct and indirect effects of atmospheric conditions and soil moisture on surface energy partitioning revealed by a prolonged drought at a temperate forest site. J. Geophys. Res..

[60] Tang Y, Wen X, Sun X, Wang H (2014). Interannual variation of the Bowen ratio in a subtropical coniferous plantation in Southeast China, 2003–2012. PLoS ONE.

[61] Lansu EM, van Heerwaarden CC, Stegehuis AI, Teuling AJ (2020). Atmospheric aridity and apparent soil moisture drought in European forest during heat waves. Geophys. Res. Lett..

[62] Falge E (2001). Gap filling strategies for defensible annual sums of net ecosystem exchange. Agric. For. Meteorol..

[63] Cleveland, W. S., Grosse, E. & Shyu, W. M. Local regression models. In *Statistical models in S* 309–376 (Routledge, 2017).

[64] Ioannidis JPA (2005). Why most published research findings are false. PLOS Med..

[65] Button KS (2013). Confidence and precision increase with high statistical power. Nat. Rev. Neurosci..

[66] Bastos A (2020). Direct and seasonal legacy effects of the 2018 heat wave and drought on European ecosystem productivity. Sci. Adv..

[67] Liu Y (2015). Water use efficiency of China’s terrestrial ecosystems and responses to drought. Sci. Rep..

[68] Yang Y (2016). Contrasting responses of water use efficiency to drought across global terrestrial ecosystems. Sci. Rep..

[69] Schnabel F (2022). Cumulative growth and stress responses to the 2018–2019 drought in a European floodplain forest. Glob. Change Biol..

[70] Mikac S (2018). Drought-induced shift in tree response to climate in floodplain forests of Southeastern Europe. Sci. Rep..

[71] Roibu C-C (2020). The climatic response of tree ring width components of ash (Fraxinus excelsior L.) and Common Oak (Quercus robur L.) from Eastern Europe. Forests.

[72] Kroll J (2022). Spatially varying relevance of hydrometeorological hazards for vegetation productivity extremes. Biogeosciences.

[73] Delpierre N (2009). Exceptional carbon uptake in European forests during the warm spring of 2007: A data-model analysis. Glob. Change Biol..

[74] Schubert P (2012). Modeling GPP in the Nordic forest landscape with MODIS time series data-Comparison with the MODIS GPP product. Remote Sens. Environ..

[75] Fu Z (2020). Sensitivity of gross primary productivity to climatic drivers during the summer drought of 2018 in Europe. Philos. Trans. R. Soc. B.

[76] Pohl F (2023). Long-term daily hydrometeorological drought indices, soil moisture, and evapotranspiration for ICOS sites. Sci. Data.

[77] Kannenberg SA, Novick KA, Phillips RP (2019). Anisohydric behavior linked to persistent hydraulic damage and delayed drought recovery across seven North American tree species. New Phytol..

[78] Li Y (2023). Widespread spring phenology effects on drought recovery of Northern Hemisphere ecosystems. Nat. Clim. Change.

[79] Zheng P (2021). Effects of drought and rainfall events on soil autotrophic respiration and heterotrophic respiration. Agric. Ecosyst. Environ..

[80] Goulden ML, Bales RC (2019). California forest die-off linked to multi-year deep soil drying in 2012–2015 drought. Nat. Geosci..

[81] Jackson RB, Moore LA, Hoffmann WA, Pockman WT, Linder CR (1999). Ecosystem rooting depth determined with caves and DNA. Proc. Natl. Acad. Sci. USA.

[82] Rocha AV, Goulden ML, Dunn AL, Wofsy SC (2006). On linking interannual tree ring variability with observations of whole-forest CO2 flux. Glob. Change Biol..

[83] Mund M (2010). The influence of climate and fructification on the inter-annual variability of stem growth and net primary productivity in an old-growth, mixed beech forest. Tree Physiol..

[84] Gielen B (2018). Ancillary vegetation measurements at ICOS ecosystem stations. Int. Agrophys..

[85] Wollschläger U (2017). The Bode hydrological observatory: A platform for integrated, interdisciplinary hydro-ecological research within the TERENO Harz/Central German Lowland Observatory. Environ. Earth Sci..

[86] Franz D (2018). Towards long-term standardised carbon and greenhouse gas observations for monitoring Europe’s terrestrial ecosystems: a review. Int. Agrophys..

[87] Rebmann C (2018). ICOS eddy covariance flux-station site setup: A review. Int. Agrophys..

[88] Aubinet M (1999). Estimates of the annual net carbon and water exchange of forests: The EUROFLUX methodology. Adv. Ecol. Res..

[89] Baldocchi D (2014). Measuring fluxes of trace gases and energy between ecosystems and the atmosphere: The state and future of the eddy covariance method. Glob. Change Biol..

[90] Kolle, O. & Rebmann, C. *EddySoft: Documentation of a Software Package to Acquire and Process Eddy Covariance Data*, vol. 2007 (MPI-BGC, 2010).

[91] Fratini G, Mauder M (2014). Towards a consistent eddy-covariance processing: An intercomparison of EddyPro and TK3. Atmos. Meas. Tech..

[92] Mauder, M., Foken, T., Aubinet, M. & Ibrom, A. Eddy-covariance measurements. In *Springer Handbook of Atmospheric Measurements* 1485–1515 (Springer, 2021).

[93] Wutzler T (2018). Basic and extensible post-processing of eddy covariance flux data with REddyProc. Biogeosciences.

[94] Montagnani L (2018). Estimating the storage term in eddy covariance measurements: the ICOS methodology. Int. Agrophys..

[95] Papale D (2006). Towards a standardized processing of Net Ecosystem Exchange measured with eddy covariance technique: Algorithms and uncertainty estimation. Biogeosciences.

[96] Reichstein M (2005). On the separation of net ecosystem exchange into assimilation and ecosystem respiration: review and improved algorithm. Glob. Change Biol..

[97] Lasslop G (2010). Separation of net ecosystem exchange into assimilation and respiration using a light response curve approach: critical issues and global evaluation. Glob. Change Biol..

[98] Lloyd J, Taylor JA (1994). On the temperature dependence of soil respiration. Funct. Ecol..

[99] Filippa G (2016). Phenopix: A R package for image-based vegetation phenology. Agric. For. Meteorol..

[100] Gillespie AR, Kahle AB, Walker RE (1987). Color enhancement of highly correlated images. II. Channel ratio and “chromaticity” transformation techniques. Remote Sens. Environ..

[101] Sonnentag O (2012). Digital repeat photography for phenological research in forest ecosystems. Agric. For. Meteorol..

[102] Klosterman ST (2014). Evaluating remote sensing of deciduous forest phenology at multiple spatial scales using PhenoCam imagery. Biogeosciences.

[103] Kline, M. *Calculus: An Intuitive and Physical Approach* (Courier Corporation, 1998). Google-Books-ID: YdjK_rD7BEkC.

[104] Vicente-Serrano SM, Beguería S, López-Moreno JI (2010). A multiscalar drought index sensitive to global warming: The standardized precipitation evapotranspiration index. J. Clim..

[105] Wu D (2015). Time-lag effects of global vegetation responses to climate change. Glob. Change Biol..

[106] Yu P (2022). Interannual variation of gross primary production detected from optimal convolutional neural network at multi-timescale water stress. Remote Sens. Ecol. Conserv..

[107] Stone, C. J. & Koo, C.-Y. Additive Splines in Statistics. *Proceedings of the American Statistical Association* 5 (1985).

[108] Gauthier J, Wu QV, Gooley TA (2020). Cubic splines to model relationships between continuous variables and outcomes: a guide for clinicians. Bone Marrow Transplant..

[109] Durrleman S, Simon R (1989). Flexible regression models with cubic splines. Stat. Med..

[110] Harrell, F. E. *Regression Modeling Strategies: With Applications to Linear Models, Logistic and Ordinal Regression, and Survival Analysis. Springer Series in Statistics* (Springer International Publishing, Cham, 2015).

