# Supplementary material for: Observational evidence of legacy effects of the 2018 drought on a mixed deciduous forest in Germany
Source: Sci Rep. 2023 Jul 5;13:10863. doi: 10.1038/s41598-023-38087-9 (PMC10322863; doi:10.1038/s41598-023-38087-9)
Supplement: Supplementary file 1 — Supplementary Information. [file 41598_2023_38087_MOESM1_ESM.pdf]

Supplementary material

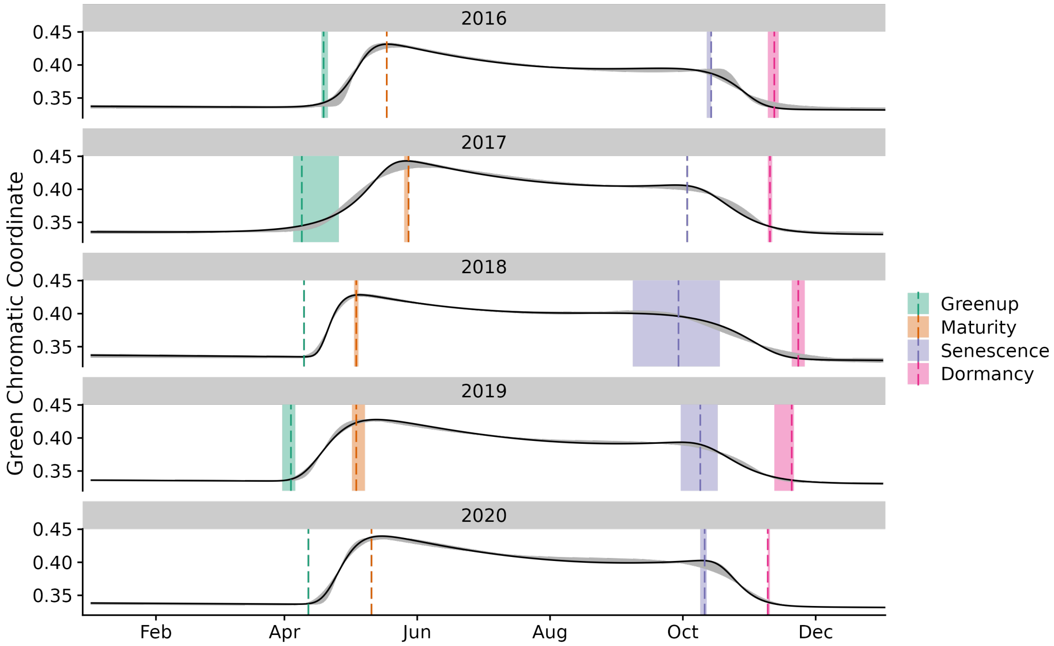

Figure S1. Time series of green chromatic coordinate (GCC) extracted from Phenocam pictures and dates of phase transition.

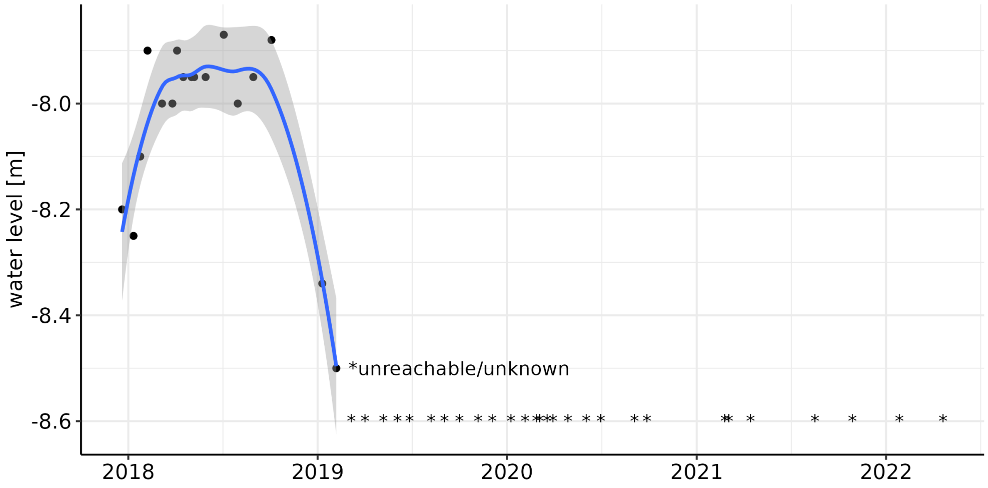

Figure S2. Measurements of groundwater level at the research site with an electric contact gauge. From early 2019, the instruments could no longer reach the water level.
